# Inhibition of Fas-Associated Death Domain-Containing Protein (FADD) Protects against Myocardial Ischemia/Reperfusion Injury in a Heart Failure Mouse Model

**DOI:** 10.1371/journal.pone.0073537

**Published:** 2013-09-13

**Authors:** Qian Fan, Zheng M. Huang, Matthieu Boucher, Xiying Shang, Lin Zuo, Henriette Brinks, Wayne Bond Lau, Jianke Zhang, J. Kurt Chuprun, Erhe Gao

**Affiliations:** 1 Center for Translational Medicine, Temple University School of Medicine, Philadelphia, Pennsylvania, United States of America; 2 Department of Gerontology, Beijing Chaoyang Hospital-Affiliate of Beijing Capital Medical University, Beijing, China; 3 Department of Emergency Medicine, Thomas Jefferson University Hospital, Philadelphia, Pennsylvania, United States of America; 4 Department of Microbiology and Immunology, Thomas Jefferson University, Philadelphia, Pennsylvania, United States of America; Virginia Commonwealth University Medical Center, United States of America

## Abstract

**Aim:**

As technological interventions treating acute myocardial infarction (MI) improve, post-ischemic heart failure increasingly threatens patient health. The aim of the current study was to test whether FADD could be a potential target of gene therapy in the treatment of heart failure.

**Methods:**

Cardiomyocyte-specific FADD knockout mice along with non-transgenic littermates (NLC) were subjected to 30 minutes myocardial ischemia followed by 7 days of reperfusion or 6 weeks of permanent myocardial ischemia via the ligation of left main descending coronary artery. Cardiac function were evaluated by echocardiography and left ventricular (LV) catheterization and cardiomyocyte death was measured by Evans blue-TTC staining, TUNEL staining, and caspase-3, -8, and -9 activities. In vitro, H9C2 cells transfected with ether scramble siRNA or FADD siRNA were stressed with chelerythrin for 30 min and cleaved caspase-3 was assessed.

**Results:**

FADD expression was significantly decreased in FADD knockout mice compared to NLC. Ischemia/reperfusion (I/R) upregulated FADD expression in NLC mice, but not in FADD knockout mice at the early time. FADD deletion significantly attenuated I/R-induced cardiac dysfunction, decreased myocardial necrosis, and inhibited cardiomyocyte apoptosis. Furthermore, in 6 weeks long term permanent ischemia model, FADD deletion significantly reduced the infarct size (from 41.20±3.90% in NLC to 26.83±4.17% in FADD deletion), attenuated myocardial remodeling, improved cardiac function and improved survival. In vitro, FADD knockdown significantly reduced chelerythrin-induced the level of cleaved caspase-3.

**Conclusion:**

Taken together, our results suggest FADD plays a critical role in post-ischemic heart failure. Inhibition of FADD retards heart failure progression. Our data supports the further investigation of FADD as a potential target for genetic manipulation in the treatment of heart failure.

## Introduction

Dramatic advances in the past 30 years have improved the management of ischemic heart disease and acute myocardial infarction (MI). As a result, mortality from coronary artery disease and acute MI has declined, although congestive heart failure (CHF) only reduced to a much lesser extent [1]. Over the last 25 years, hospitalizations with CHF as the principal or secondary diagnosis in patients 65 years or older have actually increased by 70–100% [2]. As increased patient survival from initial MI continues, the population now subject to MI's clinical sequelae, including heart failure, grows [3].

Acute MI may not necessarily result in left ventricular (LV) dysfunction and heart failure, a process dependent upon several factors, including myocardial stunning, hibernation, and remodeling, as well as neuroendocrine activation. Although the precise cellular and molecular basis underlying complex heart failure pathogenesis is not understood completely, involvement of cardiomyocyte death, particularly apoptosis, is a pivotal element [4], [5].

Ubiquitously inherent within metazoan cells, apoptosis is programmed suicide [6]. The involved death machinery may be activated by stimuli originating outside (e.g., nutrient/oxygen/survival factor deficiency, reactive oxygen species, stretch, ultraviolet radiation, drugs) or inside (e.g., cell cycle perturbations, deoxyribonucleic acid damage) [7]. Two types of apoptosis contribute to post-ischemic heart failure progression [8]–[10]. Firstly, a high-frequency fleeting burst of cell death occurs within the first 24 hours in the infarct zone. Secondly, infrequent myocyte apoptosis may persist for months in the remote myocardium. Mitochondria and death receptors (DR) mediate the two major apoptotic pathways [10]. Evidence suggests that the DR pathway has more involvement with the remote, persistent myocardial apoptotic subtype. Fas-associated death domain-containing protein (FADD) is a vital mediator of the DR pathway [11]. FADD is involved with DR signaling initiated by Fas, TNFR-I, TRAIL receptors (or DR4/5), and DR3 [12]. FADD contains two protein-protein interaction structures: the death domain (DD) at the carboxy terminus and the death effector domain (DED) at the amino terminus. The DD of FADD binds to the corresponding domain within the intracellular tail of Fas, whereas the DED of FADD associates with its respective domain in procaspase-8 [13], [14]. Apoptotic signaling is initiated by Fas clustering induced by trimeric Fas ligand (FasL) engagement, a signaling complex formation containing FasL, Fas, FADD, and procaspase-8 [15]. Procaspase-8 aggregation within the death-inducing signaling complex facilitates its autoproteolysis, leading to its activation as a cysteine protease. Caspase-8 then activates downstream caspases, leading to apoptotic cell death. Interacting with DED, cellular FLICE-inhibitory protein (cFLIP) showed critical role in the regulation of tumour necrosis factor mediated apoptotic signaling pathway [16], [17], while it protected against the development of post-infarction cardiac remodeling in mice through interrupting JNK1/2 signalling and augmenting Akt signalling [18].

Given known the great importance of FADD signaling pathway, we hypothesize FADD may represent a promising gene target for therapeutic manipulation in attenuating post-ischemia heart failure development. The aims of the current study are: (1) to determine whether FADD deletion may promote cardiac function recovery during the acute phase after myocardial ischemia/reperfusion (I/R); (2) to determine whether FADD deletion may rescue cardiomyocytes from cell death during the acute phase after I/R; (3) to determine whether FADD deletion may delay long-term ischemia induced heart failure; and (4) to determine whether FADD deletion may decrease long-term myocardial ischemia induced cardiac tissue death.

## Methods

### Generation of cardiomyocyte-specific FADD∶GFP-deficient mice

FADD^−/−^-FADD∶GFP mice were obtained from Dr. Jianke Zhang (Department of Microbiology, Thomas Jefferson University). FADD∶GFP-deficient mice were generated as described previously [19], [20]. To generate cardiomyocyte-specific FADD-deficient mice (FADD^−/−^), MHC-Cre transgenic mice were crossed with FADD^−/−^-FADD∶GFP mice to generate FADD^−/−^-FADD∶GFP-MHC-Cre mice. Since FADD^−/−^-FADD∶GFP-MHC-Cre mice are genetically weaker compared to wild type (WT) C57/Bl6 mice we use FADD^−/−^-FADD∶GFP-MHC-Cre^−^, the non-transgenic littermates (NLC) mice line as the control.

### Experimental Protocol

Adult male cardiomyocyte-specific FADD knockout (KO, FADD^−/−^-FADD∶GFP-MHC-Cre) mice along with NLC (FADD^−/−^-FADD∶GFP) mice were anesthetized with 2% isoflurane. After exteriorizing the heart via a left thoracic incision, myocardial ischemia was induced by placing a 6-0 silk slipknot around the left main descending coronary artery as previously described [21], [22]. After 30 minutes of ischemia, the slipknot was released, and the myocardium was reperfused for 3 hours (for TUNEL and caspase activity assays), 24 hours (for echocardiographic, hemodynamic, and infarct size assays) and 7 days (echocardiographic, hemodynamic, and TUNEL assays). To determine the long term effects of FADD deletion, a subset of mice were subjected to permanent myocardial ischemia (MI), and underwent echocardiographic measurement at baseline, 2, 4, and 6 weeks after MI. After 6 weeks MI, hemodynamic assays commenced, and cardiectomies were performed for TTC staining. For survival study, mice were followed up for 60 days after sham or MI. All experiments were carried out according to the National Institutes of Health Guidelines on the Use of Laboratory Animals and all procedures were approved by the Animal Care Committee at Thomas Jefferson University and Temple University.

### Western Blotting

Heart tissue samples were homogenized in ice-cold lysis buffer. After homogenization, the lysates were centrifuged, and supernatant was saved and separated by electrophoresis on SDS-PAGE and transferred onto polyvinylidene difluoride (PVDF)-plus membranes. After blocking buffer, the immunoblots were probed with anti-FADD (Santa Cruz Biotechnology, CA) and anti-GAPDH antibodies overnight at 4°C, followed by incubation with fluorescent conjugated secondary antibodies at room temperature for 1 hour.

### Cardiac function measurement

Cardiac function was determined by echocardiography (VisualSonics VeVo 770 imaging system) and by hemodynamic measurement via LV catheterization (1.2-Fr micromanometer, Millar Instruments, Houston, Texas) at above mentioned time points. Both methods have been described in detail in our previous publications [21], [23], [24].

### Cardiomyocyte death (infarct size and apoptotic ratio)

#### Infarct size determination

Infarct size was determined by Evans blue-TTC staining after I/R as previously described [21], [22], [25]. In brief, after I/R, the ligature around the coronary artery was retied and 0.05 ml of 1% Evans blue dye was injected into the left coronary artery (LCA). The heart was quickly excised and frozen for 1 hour at −80°C. Each heart was then sectioned into 1.0 mm portions, and incubated at 37°C in 1% TTC-PBS for 15 minutes. Each stained cardiac section was photographed and analyzed using the computer-based image analyzer SigmaScan Pro 5.0 (SPSS Science, Chicago, IL). The areas were defined as follows: the infarct area (Inf) consists of the TTC-negative staining region, the area at risk (AAR) consists of the Evan's Blue negatively staining region – including the TTC-positive staining and TTC-negative staining regions, and the area not at risk (ANAR) or non-ischemic region consists of the Evan's blue positively staining regions. Myocardial infarct size was calculated as a percentage of the AAR (Inf/AAR) and the AAR was calculated as the percentage of total LV (AAR/(AAR+ANAR)). In long term of MI study, the infarct size was expressed as the length of the scare/LV circumference×100.

#### TUNEL staining

Hearts were perfused with 0.1 ml PBS via LCA and then dissected and fixed in 4% paraformaldehyde in PBS (pH 7.4) for 24 hours at room temperature. Fixed tissues were embedded in paraffin. Five sections of 6 µm thickness were cut from each tissue block. TUNEL staining was performed per manufacturer's instructions (In Situ Cell Death Protection Kit, Fluorescein, Roche, Indianapolis, IN). Total nuclei were stained by DAPI (Vector Laboratories Inc., Burlingame, CA). Additionally, cardiac tissue was specifically labeled by α-actinin antibody. Apoptotic index (i.e., the number of positively stained nuclei/total number of nuclei counted×100%) was determined via blinded manner.

#### Caspase-3, -8, -9 activity

After 3 hours reperfusion, myocardial tissue was homogenized in ice-cold lysis buffer for 30 seconds (PRO 200 homogenizer). Homogenates were centrifuged for 5 minutes at 10,000 *g* at 4°C. Supernatants were collected, and protein concentrations were measured by BCA method (Pierce Chemical, Rockford, IL). To each well of a 96-well plate, supernatant containing 200 µg of protein was loaded and incubated with 25 µg Ac-DEVD-*p*NA, Ac-IETC-*p*NA, or Ac-LEHD-*p*NA at 37°C for 1.5 hours. *p*NA was cleaved from DEVD (by caspase 3, BIOMOL), IETD (by caspase 8, BIOMOL), or LEHD (by caspase 9, BIOMOL), and free pNA was quantified via SpectraMax-Plus microplate spectrophotometer (Molecular Devices, Sunnyvale, CA) at 405 nm. Changes in caspase activity in I/R tissue samples were calculated, and expressed as nmole *p*NA/mg/h [26]–[28].

### FADD siRNA knockdown and Immunoblotting in cell

H9C2 cells (ATCC) were cultured in DMEM (ATCC) supplemented with 10% bovine calf serum and penicillin-streptomycin in a humidified chamber with 5% CO_2_ at 37°C. Rat FADD siRNA (Origene) transient transfections were conducted by using 50 ng DNA and 3 µl HiPerfect Reagent (Qiagen) per well in 24 well plates. Knockdown of FADD was confirmed by qualitative RT-PCR using FADD specific primers (Intergrated DNA Technologies). Real-Time quantification was performed by sybr green (Biorad) using Biorad CFR96 detection system (Biorad). Experiments were conducted on cells 48 hours after transfection. Cells were treated with 10 µM chelerythrin for 30 min and cleaved caspase-3 (CC-3) was blotted to evaluate cell death.

Cells were homogenized in ice-cold RIPA buffer (50 mMol/L Tris-HCl, 135 mMol/L NaCl, 1% NP-40, 0.5% Sodium Deoxycholate, 0.1% SDS, supplemented with 1 mMol/L PMSF, 10 µg/ml Leupeptin, 20 µg/ml Aprotinin. The lysates were centrifuged at 13,000 rpm for 30 minutes at 4°C and protein concentration was determined using BCA Protein Assay (Pierce). Equal amounts of protein were t electrophoresed through 4–20% polyacrylamide gels and transferred to nitrocellulose membranes. Membranes were blocked in Odyssey Blocking Buffer (Li-COR), and then incubated with primary antibodies detecting CC-3 (Cell Signaling Technologies) and GAPDH (Santa Cruz Biotechnology) at 4°C overnight. Proteins were then detected using the Alexa Fluor 680 nm-coupled secondary antibodies (Life Technologies) using the Odyssey Infrared Imaging System (Li-COR). Quantitative densitometric analysis was performed using Odyssey infrared imaging software (version 2.1).

### Statistical analysis

All values in the text and figures are presented as mean ± SEM of n independent experiments. Statistical evaluation was performed via one-way or two-way ANOVA followed by the Bonferroni post-hoc test when appropriate. For paired comparison, T-test was used and flowered with Welch's correction. For the survival study, Kaplan-Meier analysis was used. Probabilities less than 0.05 were considered statistically significant.

## Results

### Genotyping and FADD expression pre- and post-I/R

**Figure 1:**
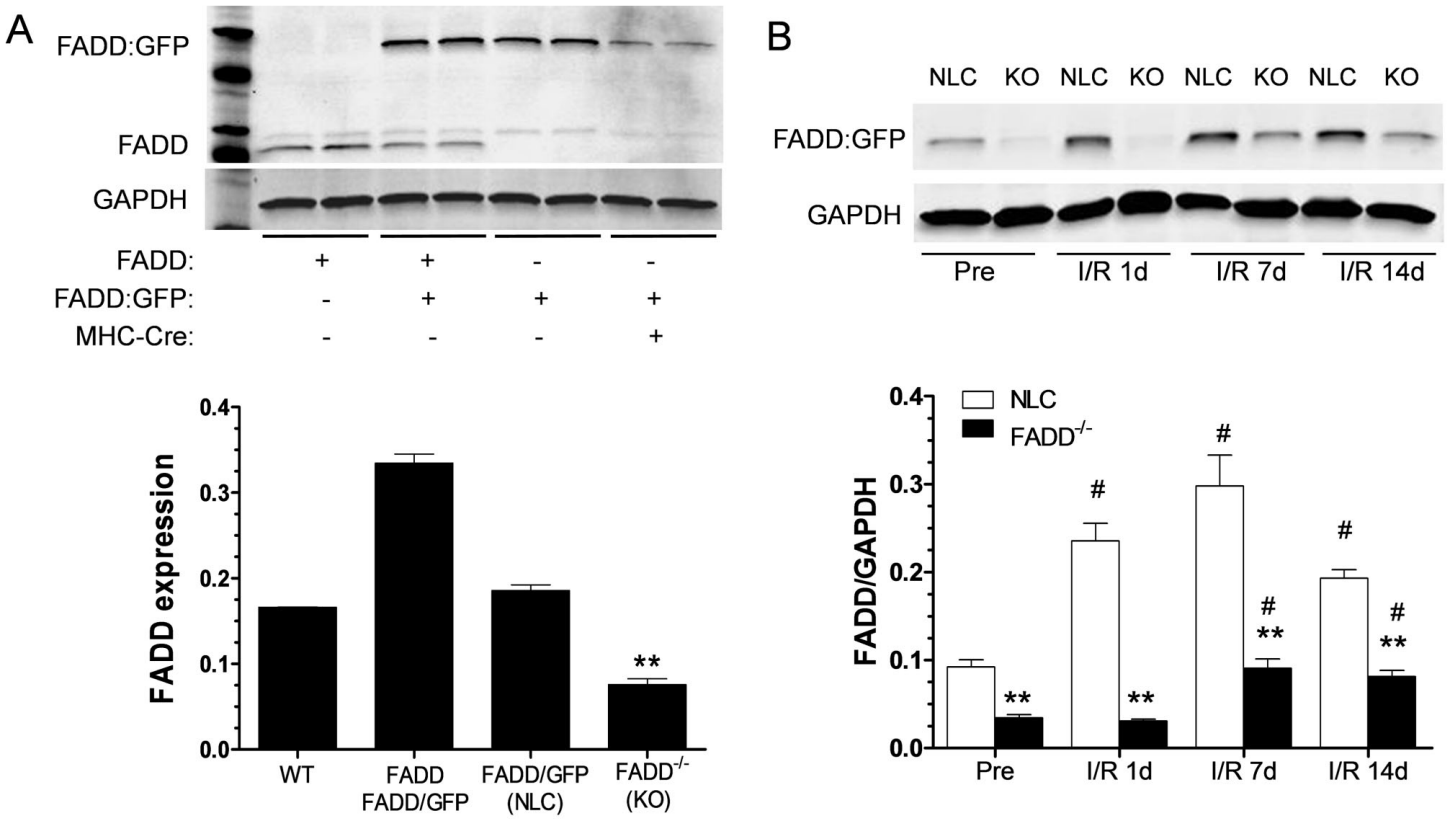
Time course of FADD expression in each group post-myocardial ischemia/reperfusion (I/R). (A) Top: Representative photomicrographs of FADD expression in cardiac tissue by western blot in WT C57/BL6 (lanes 1–2) and FADD^+/−^-FADD∶GFP-MHC-Cre^−^ (lanes 3–4) control mice, FADD^−/−^-FADD∶GFP-MHC-Cre^−^ (NLC, line 5–6) and FADD^−/−^-FADD∶GFP-MHC-Cre^+^ (FADD^−/−^, line 7–8) mice. Bottom: Ratio of FADD expression (FADD or FADD+FADD∶GFP to GAPDH). (B) Top: Representative photomicrographs of FADD∶GFP expression in cardiac tissue pre- and post-MI/R by western blot in FADD^−/−^-FADD∶GFP-MHC-Cre^−^ (NLC) and FADD^−/−^-FADD∶GFP-MHC-Cre^+^ (FADD^−/−^) groups. Bottom: Ratio of FADD expression (FADD∶GFP to GAPDH). n = 4. **P<0.01 vs. FADD^−/−^-FADD∶GFP-MHC-Cre^−^ (NLC). ^#^ *P*<0.05 vs pre-I/R condition in NLC and FADD^−/−^ groups respectively, n = 4.

Previous study from Zhang *et al.* showed that FADD∶GFP exerts similar function to FADD [19], [29]. To detect the effects of FADD upon post-ischemic heart failure, we first examined the expression level of FADD among wild type C57/Bl6 (WT, FADD^+/+^), FADD^+/−^FADD∶GFP-MHC-Cre^−^, FADD^−/−^-FADD∶GFP-MHC-Cre^−^ (NLC) and FADD^−/−^-FADD∶GFP-MHC-Cre^+^ (FADD knock out, FADD^−/−^) mouse lines as shown in Figure 1A. Compared to WT (Figure 1A, lanes 1 and 2 from the left), the level of FADD expression is significant higher in FADD^+/−^FADD∶GFP-MHC-Cre^−^ mice (lanes 3 and 4 from the left) and similar to FADD^−/−^-FADD∶GFP-MHC-Cre^−^ (NLC) line (Lanes 5 and 6 from the left), but significantly lower in FADD^−/−^-FADD∶GFP MHC-Cre^+^ mice (Lanes 7 and 8 from the left). There was no difference in FADD expression between WT (FADD^+/+^) and FADD^−/−^-FADD∶GFP-MHC-Cre^−^ (NLC) mice, but about more than 50% reduction in FADD^−/−^ mice compared to either WT or NLC mice in our whole heart tissue preparation. Additionally, we examined the level of FADD expression up to 14 days post I/R in both NLC and FADD^−/−^ groups. Ischemia/reperfusion injury increased FADD expression in both group compared to pre-I/R condition as shown in Figure 1B. However, the up-regulation of FADD was significantly less and delayed in FADD^−/−^ group. Notice that there appears to be about a 3-fold increase in FADD∶GFP levels at 7 days post I/R FADD^−/−^ group suggesting that there is an upregulation of FADD in the non-myocyte cells including endothelial cells, smooth muscle cells, and blood cells etc.

### FADD deletion improves cardiac function in the acute I/R phase

**Figure 2:**
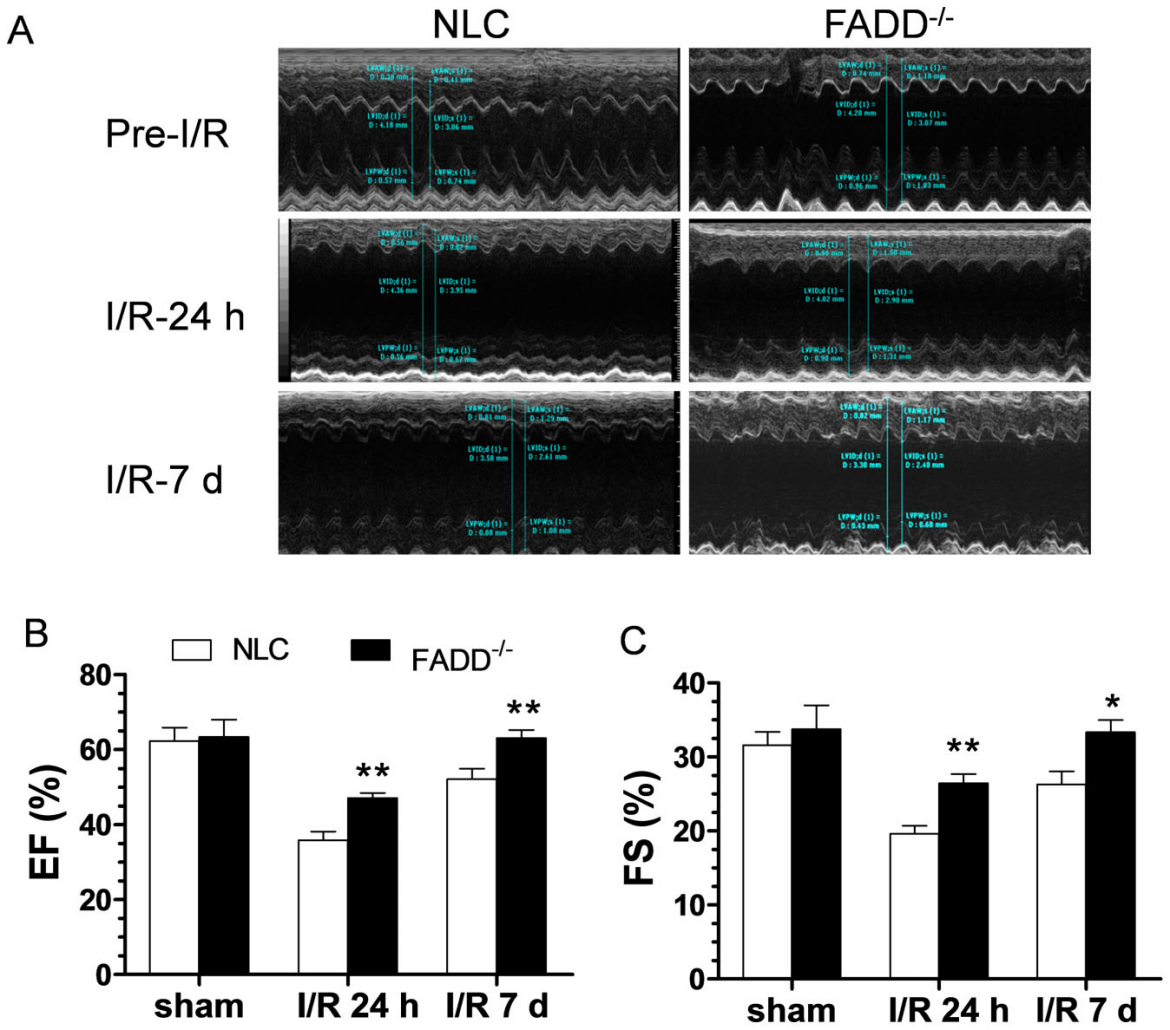
Effect of FADD^−/−^ upon cardiac function as determined by echocardiography. (A) Representative echocardiographic recordings pre- and post-24 hours and 7 days of reperfusion. (B) and (C) Graphic summary of LV ejection fraction (LVEF) and LV fractional shortening (LVFS) in groups (n = 10–14 mice/group). *P<0.05, **P<0.01 FADD^−/−^ vs. NLC control (FADD∶GFP MHC-Cre^−^).

**Figure 3:**
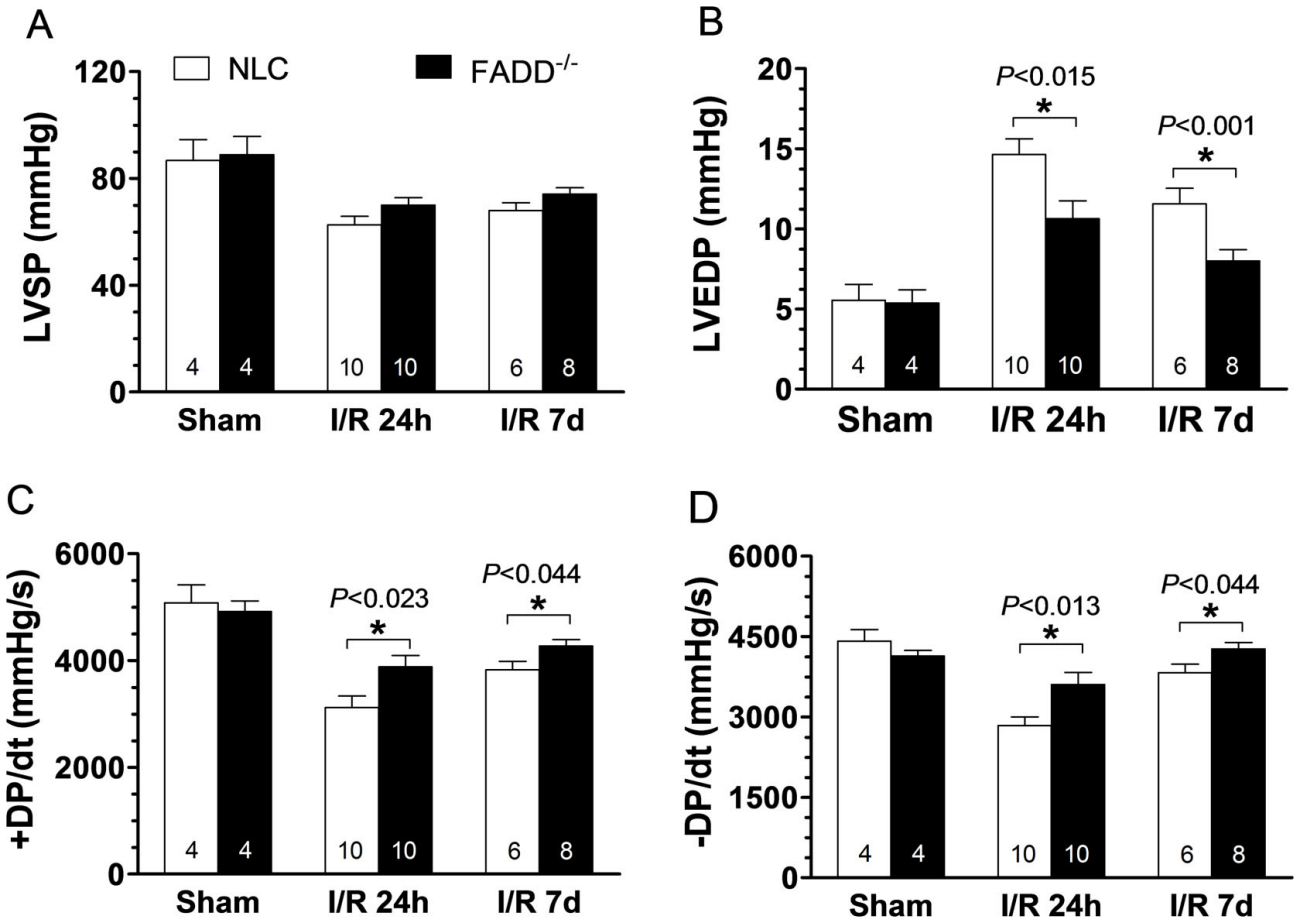
Effect of FADD^−/−^ upon cardiac function as determined by ventricular catheterization, spanning pre- to 7 days post-reperfusion. (A) Left ventricular systolic pressure (LVSP). (B) Left ventricular end diastolic pressure (LVEDP). (C) Rate of rise of left ventricular pressure (+dP/dt) and (D) Rate of reduction of left ventricular pressure (−dP/dt). n = 10–14 mice/group. *P<0.05 FADD^−/−^ vs. NLC control.

In the present study, two independent methods (echocardiography and direct ventricular catheterization) were utilized to detect potential differences in cardiac function between NLC and FADD^−/−^ mice at 2 months of age. There were no differences in body weight between NLC and FADD^−/−^ mice. Echocardiography revealed no significant differences in LV ejection fraction (EF%) and fractional shortening (FS%) between NLC and FADD^−/−^ mice before myocardial ischemia (Figure 2). However, EF% and FS% of FADD^−/−^ mice were significantly higher after 24 hours and 7 days reperfusion compared to those of NLC. Direct ventricular hemodynamic measurements were consistent with echocardiographic data. There were no significant differences between the two groups in left ventricular systolic pressure (LVSP), left ventricular end diastolic pressure (LVEDP), +dP/dt, and −dP/dt before coronary occlusion. After 24 hours and 7 days reperfusion, the values of LVEDP in FADD^−/−^ mice (10.63±1.12 and 8.0±0.71, respectively) were significantly decreased compared to NLC (14.64±0.1 and 11.57±0.97, respectively, *P*<0.015 and 0.001, Figure 3B). In addition, the values of ±dP/dt were significantly greater than that of NLC (Figure 3B and C). Thus, both echocardiographic and ventricular hemodynamic measurements demonstrated FADD deletion attenuated cardiac dysfunction during the acute I/R phase.

### FADD deletion attenuates cardiomyocyte death in the acute I/R phase

**Figure 4:**
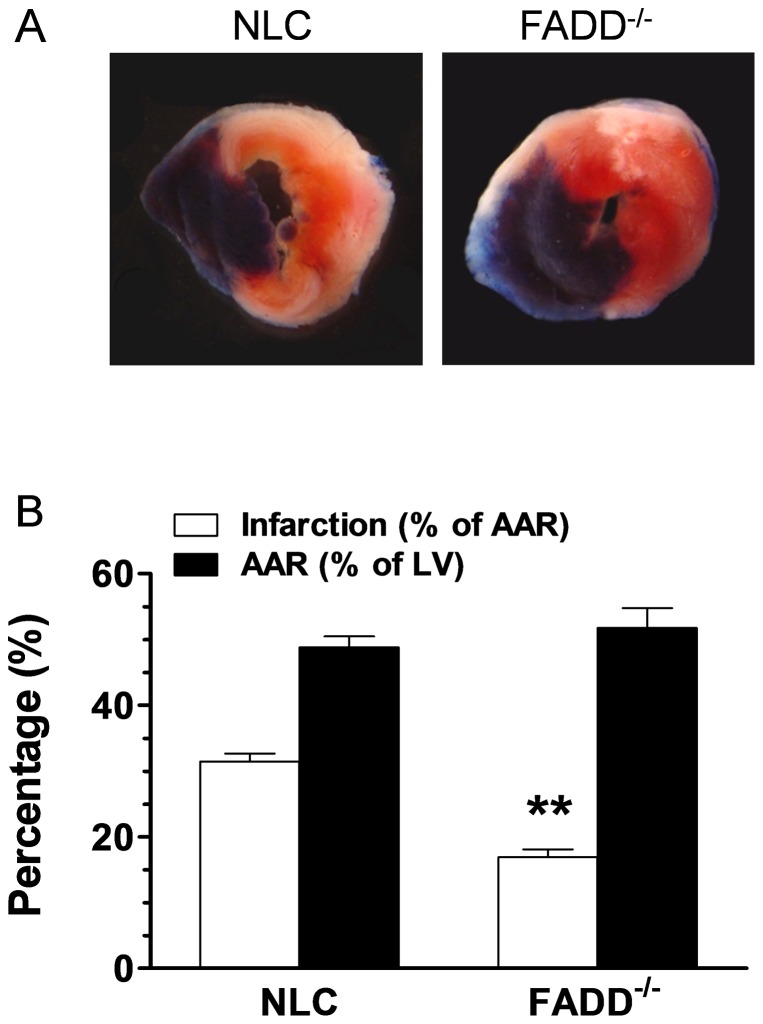
FADD^−/−^ ameliorates post-I/R myocardial infarct size. (A) Representative photograph of TTC stained heart tissue section obtained 24 hours I/R in FADD^−/−^ and NLC groups. (B) Graphic summery of infarct size expressed as percentage of area-at-risk (AAR) and the size of AAR. n = 8–10 mice/group. **P<0.01, FADD^−/−^ vs. NLC control.

**Figure 5:**
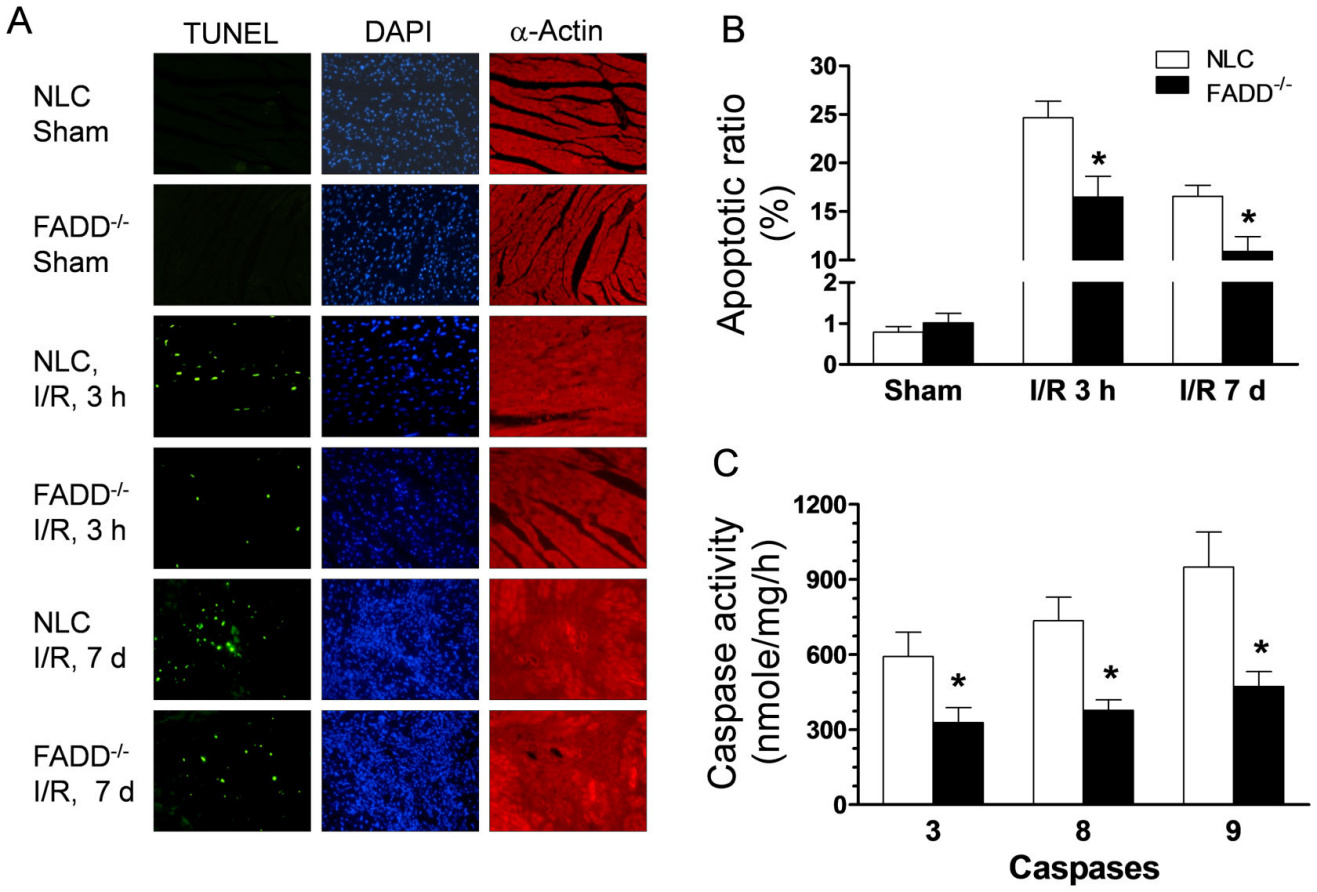
Effect of FADD^−/−^ reduced post-I/R cardiomyocyte apoptosis. (A) Representative photomicrographs of in situ detection of cardiac tissue DNA fragments from mice subjected to 30 minutes of ischemia and 3 hours or 7 days of reperfusion. Tissue sections were stained with DAPI (blue), anti-actinin (red) and TUNEL (green). TUNEL-positive nuclei were summarized in graph (B) and expressed as percentage of all tissues subject to I/R staining TUNEL-positive. (C) Caspase-3, -8, and -9 activity in ischemic cardiac tissue after 3 hours reperfusion. n = 10–12 animals/group. *P<0.05, **P<0.01 vs. NLC control.

The area of necrotic cardiac tissue (infarction) was expressed as the percentage of the area-at-risk (AAR, Figure 4). There was no significant difference between groups in the AAR expressed as the percentage of the total left ventricle area, indicating comparable degree of ischemic jeopardy in all I/R groups. After 30 minutes of coronary occlusion and 24 hours reperfusion, the FADD^−/−^ group manifested 17% infarct, compared to 31% in the NLC group as shown in Figure 4B. Consistently, FADD deletion significantly reduced TUNEL-positive staining cells after 7 days of reperfusion (Figure 5A and B). These results provided direct evidences that FADD deletion protected cardiomyocytes against the I/R injury, and suggested the involvement of FADD in I/R-induced myocardial apoptosis. Caspase-3, -8, and -9 are major contributors to the progression of I/R-induced apoptosis. Caspase-3 activation is the final common apoptotic pathway. To obtain further evidences of the anti-apoptotic effect of FADD deletion, caspase-3, -8, and -9 activities were determined in all groups. The baseline of caspase-3, -8, and -9 are less than 100 nmole/mg/h in our preparation in both NLC and FADD^−/−^ mice line. Upon the I/R challenger, all caspase-3, -8 and -9 were increased in both NLC and FADD^−/−^ groups. However, FADD knockout markedly reduced caspase-3, -8, and -9 activities compared to NLC as well (Figures 5 C).

### FADD deletion augments cardiac function after long-term myocardial ischemia

**Figure 6:**
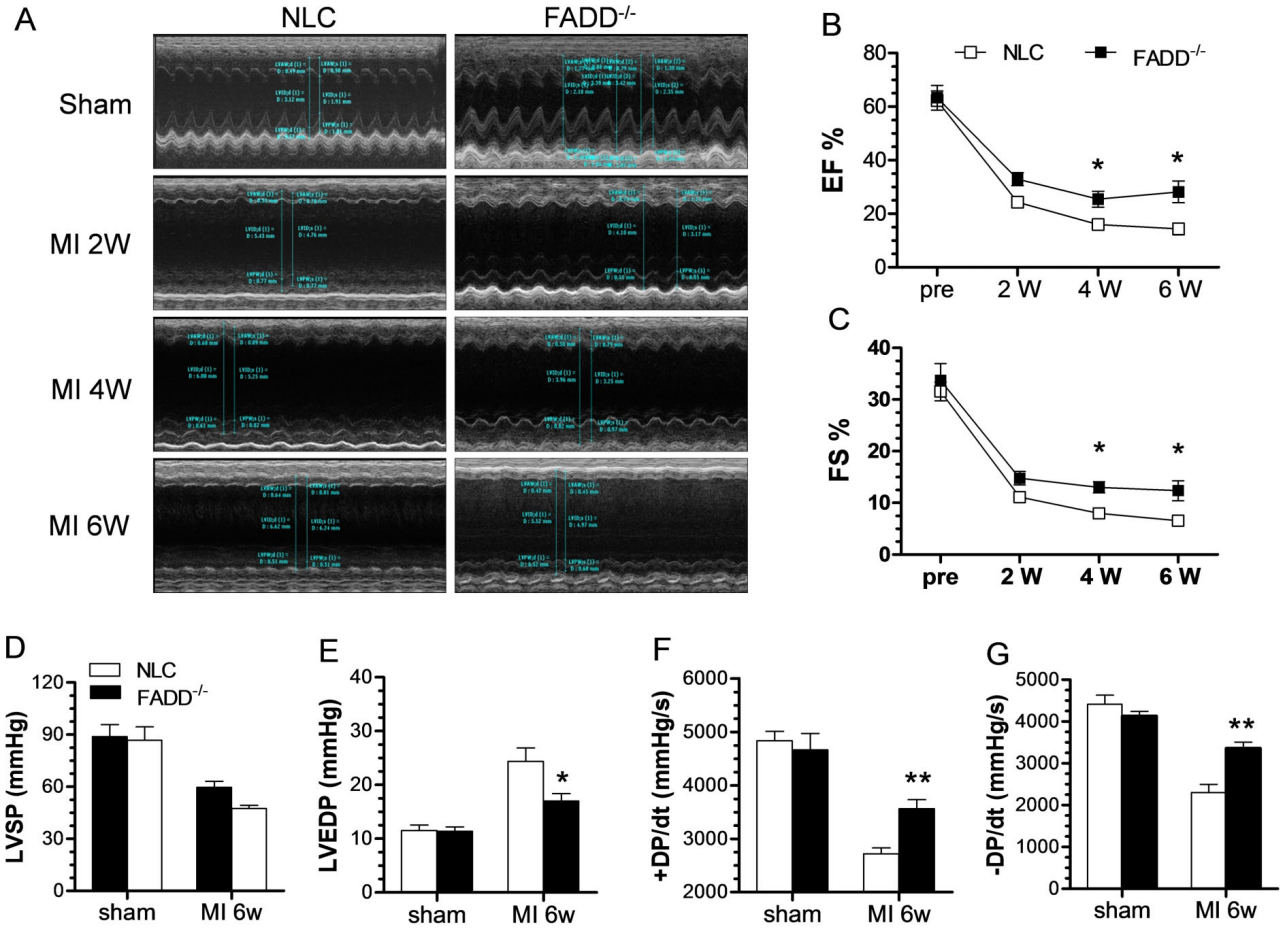
Effect of FADD^−/−^ on cardiac function after 6 weeks MI. (A) Representative photomicrographs of echocardiographic measurements in each group over 6 weeks MI. Linear chart depicts change of LVEF (B) and LVFS (C) after 6 weeks ischemia. n = 8 animals in each group. *P<0.05, **P<0.01 vs NLC (Two way ANOVA). (D–G) Hemodynamic measurements of cardiac function at 6 week post-MI or Sham in NLC and FADD^−/−^ groups. (D) LVSP, (E) LVEDP, (F) +dP/dt and (D) −dP/dt. n = 5–8 animals in each group. *P<0.05, **P<0.01 FADD^−/−^ vs. NLC control.

The above results suggest FADD deletion protects cardiomyocytes, and augments cardiac function in the acute I/R phase. We next tested a model of permanent ischemia, a six-week period of myocardial ischemia leading to post-ischemic heart failure. At baseline, no significant differences existed between groups; after ischemia, the FADD knockout group (FADD^−/−^) manifested augmented EF% and FS% via echocardiography compared to NLC after 2, 4, and 6 weeks of ischemia (Figure 6A–C). These results were confirmed with ventricular catheterization hemodynamic analysis (Figure 6D–G). Taken together, these data suggest that FADD deletion inhibits development of post-ischemic heart failure.

### FADD deletion attenuates long-term myocardial ischemia induced myocardial death and remodeling

**Figure 7:**
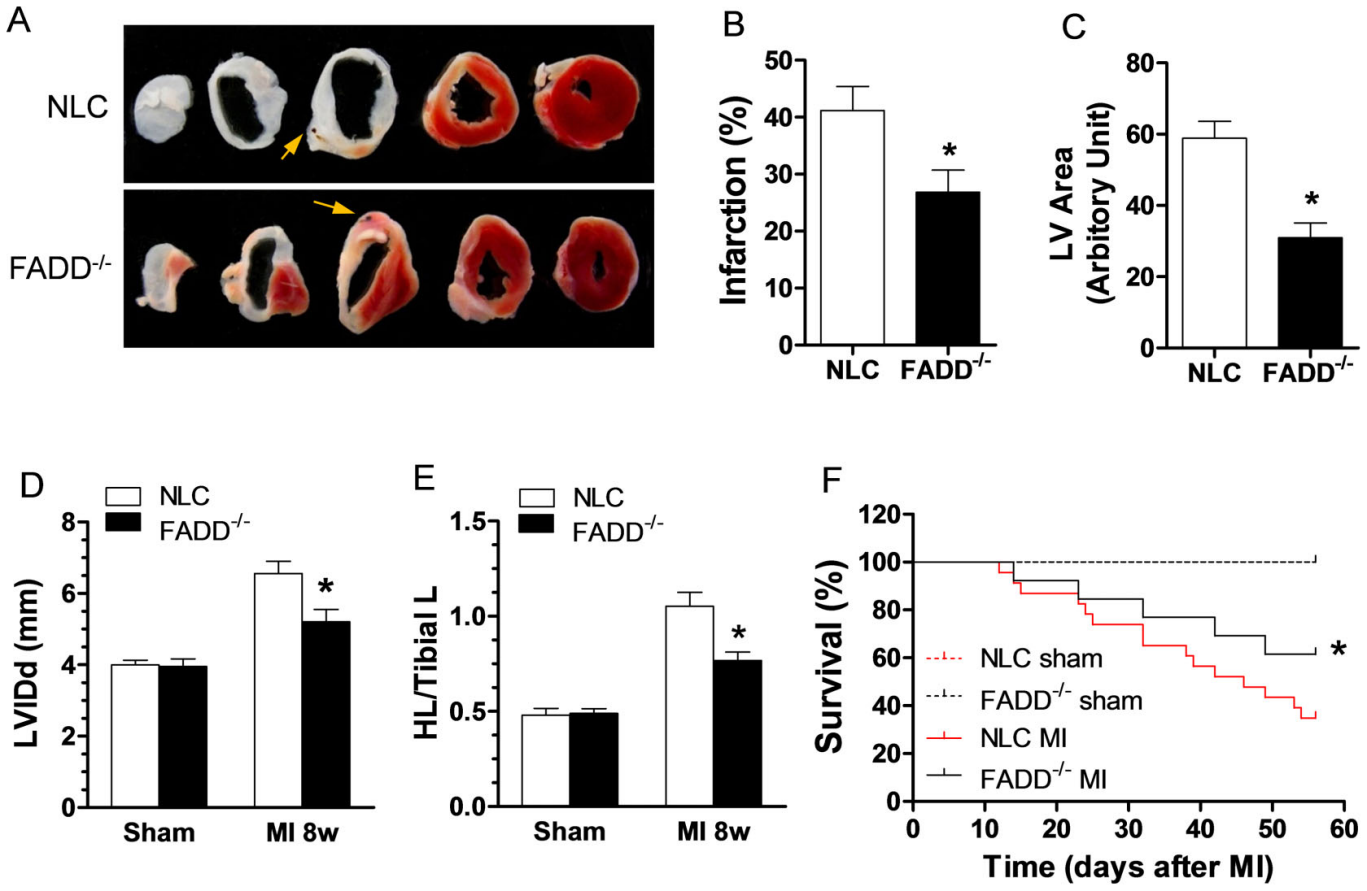
Effect of FADD^−/−^ on cardiac infarct size, cardiac remodeling and survival after 6 weeks MI. (A) representative TTC stained heart tissue section at 6 week post-MI in FADD^−/−^ and NLC control groups. (B) Graphic summary of infarct size expressed as the length of the scare/LV circumference, n = 8 animals in each group. (C) Graphic presentation of LV area in NLC and FADD^−/−^ groups, *P*<0.05 FADD^−/−^ vs. NLC, n = 8 in each groups. (D) Graphic presentation of LVIDd measured by echocardiography. *P*<0.05 FADD^−/−^ vs. NLC, n = 8 in each groups. (E) Graphic presentation the ratio of heart length (HL)/tibia length (TL) in sham or MI mice. *P*<0.05 FADD^−/−^ vs. NLC, n = 8 in each groups. (F) Survival curve in 8 week post-sham (n = 8 in each group) or post-MI mice (n = 23 in each group). *P<0.05, FADD^−/−^ vs. NLC control.

TTC staining (as described above) was utilized to determine long-term myocardial ischemia-induced cardiomyocyte death. After 6 weeks of coronary occlusion, the FADD^−/−^ group manifested 26.83±4.17% infarct, compared to 41.20±3.90% in the NLC group. A representative photograph is presented in Figure 7A, and yellow arrows point to the suture knots where the coronary artery blood flow was interrupted. The results demonstrated that, even with similar level of myocardial ischemia, infarct size in FADD^−/−^ group was significantly smaller than in the NLC group. In addition, after 8 weeks of coronary occlusion, hearts underwent significant remodeling by increasing LV volume and size in both groups; however, the remodeling was significantly less in FADD^−/−^ group compared to NLC control mice (Figure 7C and D). The ratio of heart length/tibia length (HL/TL) was about 0.5 in both sham groups, and 1.053±0.31 and 0.77±0.13 in the NLC or FADD^−/−^ MI groups, respectively (Figure 7E). Consistent with those results, FADD^−/−^ group had a better survival rates compared to NLC control (Figure 7F). Taken together, these data supports the fact that FADD deletion attenuates cardiac death in permanent coronary occlusion (long-term ischemia) model.

### FADD knockdown attenuates chelerythrin-induced apoptosis in cell

**Figure 8:**
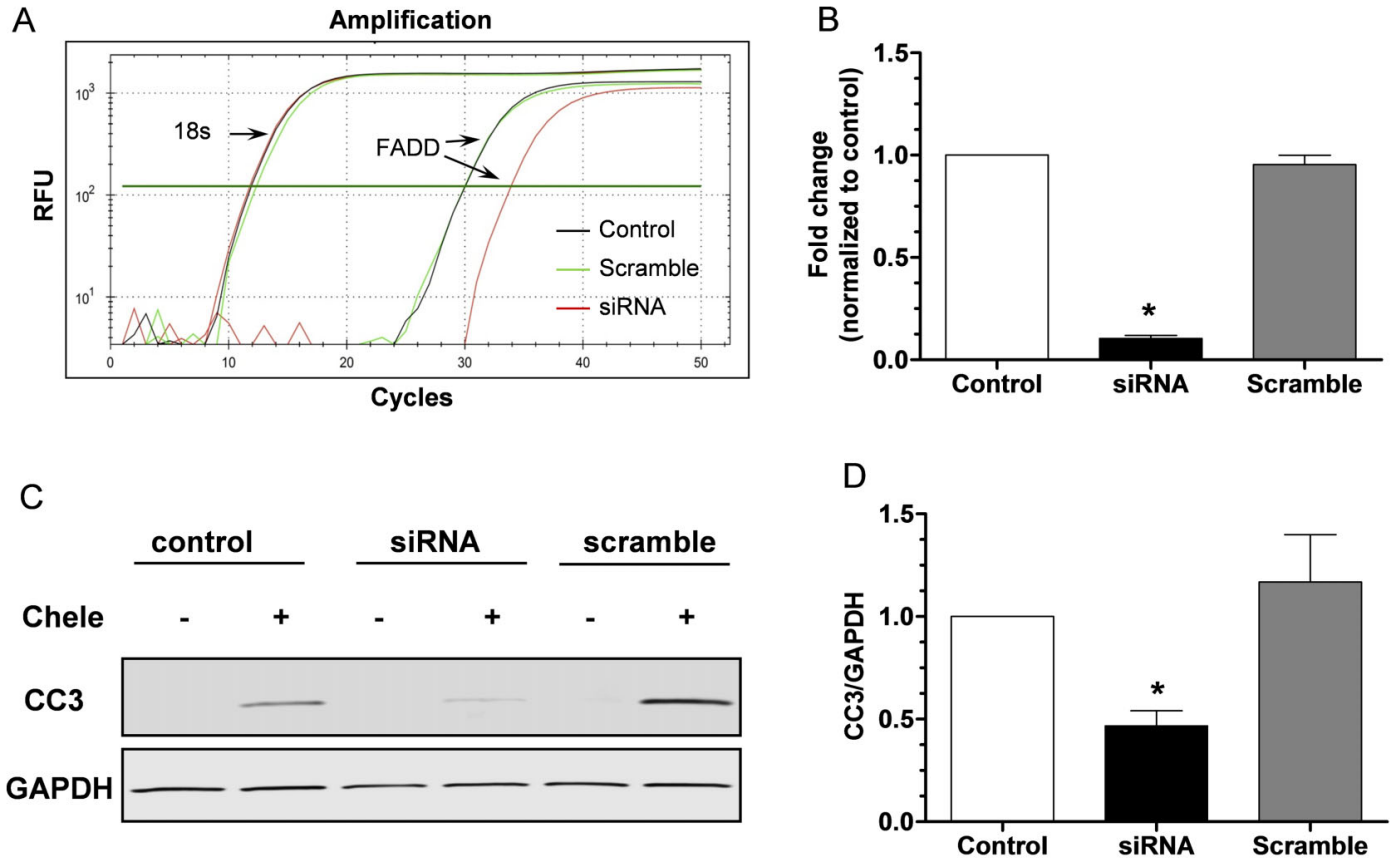
Knockdown FNDD in cell reduces chelerythrin-induced apoptosis. (A) Representative real time PCR tracings of transcript for 18S and FADD in H9C2 cells. Black: control, green: scramble siRNA, and red: FADD siRNA. (B) Quantification of relative mRNA expression of FADD in FADD specific siRNA or scrambled siRNA transfected H9C2 cells compared to control, *P<0.05, *t* test, n = 4 per group. (C) Representative Western blot showing the release of cleaved caspase-3 (CC-3) in H9C2 cells treated with chelerythrin. (D) Quantification of CC-3 release in FADD specific siRNA or scrambled siRNA transfected H9C2 cells compared to that of control cells, *P<0.05, ANOVA, n = 4 per group.

To confirm the critical role of FADD mediated cell death we knockdown FADD in H92C cell via the siRNA transfection method as we previously described [30]. Treating the cell with siRNA significantly decreased FADD expression by real time PCR as show in Figure 8 A and B. There was no difference in 18 s expression indicating the specific knockdown to FADD (Figure 8A). As we expected, challenge the cell with chelerythrin significantly reduced CC-3 expression in FADD siRNA treated cell group (Figure 8 C and D) indicating the amelioration of apoptosis with FADD knockdown.

## Discussion

The present study makes several novel discoveries. Firstly, FADD deletion inhibited cardiomyocyte apoptosis following acute I/R. The anti-apoptotic effects of FADD deletion were mediated not only by the death receptor pathway, but also the mitochondrial pathway (as demonstrated by the reduced caspase-9 activity). Secondly, FADD deletion improved cardiac function after I/R. Finally, and most importantly, FADD deletion attenuated long term-ischemia induced cell death, and delayed post-ischemic heart failure progression. Although the anti-apoptotic effects of FADD inhibition have been recognized for more than a decade, to our knowledge, this is the first study demonstrating genetic manipulation of FADD for successful amelioration of post-ischemic heart failure.

The earliest gene therapies involving FADD centered on anti-tumor and immunomodulative therapies. Kondo *et al.* investigated the effect of FADD overexpression in malignant glioma progression, revealing that regardless of Fas/APO-1 expression levels, FADD gene overexpression significantly inhibited both *in vitro* and *in vivo* survival of malignant glioma cells via apoptosis induction [31]. In another study, Kobayashi *et al.* eliminated synoviocytes via injection of local FADD-expressing adenovirus (Ad-FADD), inducing apoptosis of rheumatoid synovium, demonstrating the clinical possibilities of FADD manipulation [32], [33]. FADD has therefore been an attractive possible gene therapy target in the treatment of various diseases involving apoptotic mechanisms, including oncologic, autoimmune, and possibly cardiovascular pathologies. In the present study, we utilized cardiac-specific FADD^−/−^ mice and demonstrated that FADD deletion improved post-ischemic cardiac function and alleviated post-ischemia cardiomyocytes apoptosis. This anti-apoptotic effect of FADD deletion was further confirmed in FADD siRNA infected cell study. Furthermore, in a permanent ischemia model, FADD deletion postponed post-ischemic heart failure phenotype development and improved survival.

Existing evidence strongly suggests an important pathophysiological role for the death receptor apoptotic pathway in heart failure pathogenesis. The best-characterized death receptors are Fas (also termed CD95 or Apo1) and tumor necrosis factor receptor 1 (TNFR1). Ferrari *et al.* and Testa demonstrated patients with end-stage congestive heart failure harbored elevated circulating levels of TNF-α (ligand for TNFR1) and studies suggest a direct relationship between serum TNF-α levels and heart failure severity [34], [35]. Additionally, both Fas and FasL expression were increased in hypoxic myocytes, and Fas pathway activation has been shown to induce cardiomyocyte apoptosis [36], [37]. Consistent with these reports, our study supports that FADD expression is increased dramatically in ischemic heart tissue. However, in FADD^−/−^ mice, the post ischemia induced up regulation of FADD was inhibited to a much less degree. Previous studies and our results support the activation of the death receptors pathway after ischemia/reperfusion, evidenced by activated FADD expression. The direct result of activated FADD is apoptosis mediated by increased caspase-8 activity and downstream caspase-3 activity [14]. FADD deletion attenuates cardiomyocyte death and improves cardiac function, ultimately protecting the ischemic heart from heart failure.

We demonstrated that FADD^−/−^ decreased not only caspase-3 and -8 activities, but also caspase-9 activity. Several studies investigating cross talk between the apoptotic pathways explain this phenomenon. Date *et al.* demonstrated that FasL overexpression activated both caspase-8 and -9 in neonatal cardiomyocytes [38]. Recently, receptor-interacting protein kinases-1 (RIPK1) and RIPK3 as a lethal defect in caspase-8-, FADD-, and FLIP-deficient animals and tissues has been reported [39]. The RIPKs are known as killers, being responsible for a nonapoptotic form of cell death with features similar to necrosis [40]. The present study did not investigate the underlying mechanisms of FADD^−/−^ mediated cardioprotection and possible role in necrosis pathway. However, our caspase activity results suggest FADD deletion mediate anti-apoptosis may through dual apoptotic pathways.

### Limitations

As gene therapy technology develops, identification of ideal targets ameliorating disease represents a huge challenge in the biomedical field. The current study determined whether FADD could be a therapeutic target alleviating progression of post-ischemic heart failure. Future research investigating the specific mechanisms (e.g. effects upon MAPK, Bcl-2, or relationship between FADD knockout and RIPK1 linked necrosis etc.) underlying the cardioprotective effects of FADD deletion against post-ischemic heart failure is warranted.
